# Comprehensive out-patient pulmonary rehabilitation: Treatment outcomes in early and late stages of chronic obstructive pulmonary disease

**DOI:** 10.4103/1817-1737.78420

**Published:** 2011

**Authors:** Pinar Ergün, Dicle Kaymaz, Ersin Günay, Yurdanur Erdoğan, Ülkü Yilmaz Turay, Neşe Demir, Ebru Çanak, Fatma Sengül, Nurcan Egesel, Serdal Kenan Köse

**Affiliations:** Division of Pulmonary Rehabilitation and Home Care Center, Ankara, Turkey; 1 7^th^ Chest Diseases Clinic, Atatürk Chest Diseases and Thoracic Surgery Training and Research Hospital, Ankara, Turkey; 2 Department of Biostatistics, Ankara University School of Medicine, Ankara, Turkey

**Keywords:** Chronic obstructive pulmonary disease, disease severity, functional status, pulmonary rehabilitation, shuttle walk test

## Abstract

**BACKGROUND:**

The aim was to evaluate the outcomes of a comprehensive pulmonary rehabilitation (PR) in chronic obstructive pulmonary disease (COPD) and to establish whether in early disease stage PR is as effective as in late stages of disease.

**METHODS:**

A total of 55 stable COPD patients, 28 with early and 27 with late disease stages, were assessed. Patients underwent a comprehensive out-patient PR program for 8 weeks. To eluciate the effects of PR and compare the level of improvement; lung function, dyspnea sensation [Medical Research Council (MRC)], body composition [body mass index (BMI), fat free mass (FFM), fat free mass index (FFMI)], exercise capacity [incremental shuttle walking test, endurance shuttle walking test], health related quality of life (HRQoL) with St. George Respiratory Disease Questionnaire, psycohological status (Hospital anxiety–depression (HAD) scale) were evaluated before and after PR.

**RESULTS:**

At the end of PR in the early disease stage group, the improvement in forced vital capacity (FVC) reached a statistically significant level (*P* < 0.05). In both disease stages, there were no significant differences in BMI, FFM, and FFMI. The decrease in exertional dyspnea for the two groups evaluated with the modified BORG scale were not found statistically significant, though the dyspnea scores evaluated with MRC showed significant improvements (*P* < 0.001). HRQoL and exercise capacity were significantly improved for the two groups (*P* < 0.001). Psychological status evaluated with the HAD scale improved after PR (*P* < 0.001) both in early and late stages. Gainings in the study parameters did not differ in the early and the late disease stages.

**CONCLUSIONS:**

These results showed that patients with COPD had benefited from a comprehensive PR program in an out-patient setting regardless of disease severity. Even patients with earlier stage of disease should be referred and encouraged to participate in a PR program.

Pulmonary rehabilitation(PR) is an essential component of comprehensive management of patients with symptomatic chronic obstructive pulmonary disease (COPD) and must be provided to patients who have moderate-to-severe COPD according to the global initiative for chronic obstructive lung disease (GOLD) guidelines.[1–3] It was widely accepted that COPD was a systemic disease and even with the early stages impairments in body composition, exercise capacity, and health-related quality of life can be relevant.[4–6] Pulmonary rehabilitation can change outcomes that predict survival and can improve the systemic component of COPD and its comorbidities with a potential effect on survival.[7] The appropriate selection of patients plays a key role in the success of PR.[8] Appropriate patients for pulmonary rehabilitation programs are those who recognize that their symptoms depend upon their lung disease and are motivated to be active participants in their own care to improve their health status. The only absolute contraindications are a long history of lack of compliance and unwillingness to participate. Patient referral to PR is an important issue in this point. The bulk of referrals for PR occur when patients have severe or very severe health status, and are usually categorized as stage 3 or 4, according to the GOLD. Therefore, most of the studies demonstrating the efficacy of PR have included COPD patients, especially subgroups with moderate-to-severe COPD. On the other hand, it was well known that even, when they have mild disease, to have them benefit from preventive behavior strategies, they should be referred in the early stages.[3,9–13]

Therefore, the purpose of this study was to evaluate the benefit of a comprehensive pulmonary rehabilitation program composed of exercise training, nutritional and psychosocial counseling, and educational sessions and whether patients with early stages of COPD were equally benefitted from a comprehensive PR just as in patients with late stages.

## Methods

The study was performed prospective study in a Pulmonary Rehabilitation and Home Care Unit in Atatürk Chest Diseases and Thoracic Surgery Training and Research Hospital, Ankara, Turkey in 2008.

### Patient selection

The diagnosis of COPD and the classification of severity were defined according to the global strategy for the diagnosis, management, and prevention of COPD updated in 2009.[1] All patients were suffering from dyspnea, reduced exercise tolerance, muscle deconditioning, or limitation of daily-living activities, but were in stable clinical conditions. The classification of severity was as follows: mild COPD (stage I), FEV _1_≥ 80% predicted; moderate COPD (stage II), 50% ≤ FEV1 <80% predicted; severe COPD (stage III), 30% ≤ FEV1 <50% predicted; and very severe COPD (stage IV), FEV1 < 30%. Patients with the stages of I and II grouped as early stages of COPD whereas stages III and IV were late stages in this study. Patients suffering from acute exacerbation (i.e. increased requirement for antibiotics, oral/parenteral steroids, or an increase consumption of oxygen or bronchodilators over the previous 4 weeks) and patients with lack of motivation or poor compliance, neuromuscular disorders, unstable angina, or recent (i.e., <6 months) myocardial infarction were excluded from the PR. The patients received optimal medical treatment including β_2_-agonists, anticholinergic drugs, theophylline, and/or inhaled steroids. A stable condition whilst receiving medical treatment was required before PR commenced.

### Comprehensive PR

Patients underwent an 8-week hospital based out-patient comprehensive PR and attended the rehabilitation unit on 2 half-days per week. PR consisting of: (a) verbal inputs stressing the need for adherence to therapy, (b) educational support, covering the following topics: disease education, how to control exacerbations, what pulmonary rehabilitation is?, medication advices, bronchial hygiene techniques, and breathing control techniques, energy conservation, relaxation, and dietary advices. Educational sessions were delivered by two chest physicians, two physical therapists, a dietician, two respiratory nurses, and a psychologist. Each educational program was 45 min long and repeated every month, (c) exercise training, (d) a nutritional intervention, and (e) psychological counseling, if needed. The rehabilitation program was completely tailored to suit the needs of the individual. According to guideline recommendations, the exercise program was also tailored to the individual and a group of exercises was chosen for each patient according to their ability to tolerate exercise and their disease severity.[8] Exercise included cycle ergometer training (15 min), treadmill training (15 min), upper and lower extremity strength training (5–10 min), breathing therapies (10–20 min), and relaxation therapies (5–10 min) for total 50–70 min/day. Patients underwent both cycle ergometer and treadmill training. Both workload for cycling and walking speed for treadmill ergometer were calculated from incremental shuttle walking test (ISWT) results using formulations and BORG dyspnea scores (4–6) were also used for prescribing exercise.[5,8] Patients were trained at 50% of peak workload and 50–80% of peak VO_2_. Exercises intensity increased according to the patient progress. The supervised exercise training was implemented with the patient to physical therapists ratio of 3/1. Pulse oximetry was used to supervise patients during exercise. If the SpO_2_ fell below 90%, oxygen supplementation was provided to maintain SpO_2_ ≥ 90%. All of the patients received an identical but if necessary individualised education program consisted of eight sessions of seminars and discussions.

### Outcome measures

The outcome measures were dyspnea sensation, exercise capacity, HRQoL, body composition, and psychological status. Spirometry performed according to guidelines.[1,14] Dyspnea was assessed by MRC and modified BORG dyspnea scales.[8,9,15,16] Exercise capacity was evaluated with ISWT and endurance shuttle walking test (ESWT).[17,18] Health status-HRQoL was assessed using St. George Respiratory Questionaire (SGRQ).[4,19]

Body length was measured to the nearest 0.5 cm while the subjects were barefoot and standing (WM 715; Lameris, Breuke-len, The Netherlands). The main variables of interest for body composition were body mass index (BMI), fat free mass (FFM), and fat free mass index (FFMI). BMI was calculated as weight (kg)/height square (m^2^). FFM was measured as previously described by bioelectrical impedance analysis (BIA model TBF-300) with an operating frequency of 50 kHz at 500 μA.[20] FFM was standardized for height and expressed the FFMI [FFM (kg)/height square (m^2^)].[21]

Hospital anxiety depression (HAD) scale was used for psychometric assessment.[22] All measurements were assessed at admission and at the end of the PR.

### Statistical analysis

Statistical analyses were performed using the SPSS 15.0 (SPSS, Chicago, IL, USA). Descriptive statistics were performed for all the recorded variables. Baseline characteristics between the two groups (early vs. late stage) were compared by means of unpaired *t*-test. To compare categorized variables between early and late stage groups, the Mann–Whitney *U*-test was used. Nonparametric paired variables between before and after PR were analyzed with the Wilcoxon test. Results are shown as change between post-treatment and baseline levels (Δ values). For SGRQ analyses were performed on the total as well as subscale scores. Threshold for statistical significance was set at 0.05.

## Results

### Baseline characteristics

Between September 2007 and November 2008, 55 COPD patients who participated in our outpatient PR program (for a period of 8 weeks) were recruited in this study. They were grouped according to the severity of disease stages as early (GOLD stages I + II; *n* = 28) and late disease stages of COPD (GOLD stages III + IV; *n* = 27). The baseline characteristics of the patients are shown in Table 1. The mean age of patients in early stage was 63.25 ± 10.10 whereas it was 62.81 ± 7.18 in late stage (*P* = NS). The mean value for dyspnea sensation was significantly higher in the late disease stage when evaluated with the MRC scale (*P* = 0.05). Measures of nutritional status in the meaning of BMI (*P* = 0.001), FFM (*P* = 0.05), and FFMI (*P* = 0.01) were significantly different among the two groups. Patients in the early stage walked 63.45 m longer during the ISWT, but the difference was not significant. Exercise endurance time was not also significant between the two groups. In the case of SGRQ, scores for total, activity, and symptom were impaired more in the late disease stage, though the differences were not statistically significant. Psychometric assessments with HAD questionnaire revealed similar impairment levels in both groups (*P* = NS).

**Table 1:** Baseline characteristics (mean ± SD) of the two groups of COPD patients

Parameters	Early stage (GOLD; I + II)	Late stage (GOLD; III + IV)	*P* value
Number	28	27	
Age, year	63.25 ± 10.10	62.81± 7.18	0.07
FVC (% predicted)	58.17 ± 19.82	49.70 ± 13.20	0.09
FEV1 (% predicted)	42.46 ± 17.40	27.33 ± 8.20	**0.001**
FEV1/FVC	63.42 ± 11.42	39.85 ± 6.84	**0.001**
MRC	3.10 ± 1.13	3.81 ± 0.83	**0.05**
Exercise-BORG	2.85 ± 1.20	3.40 ± 0.88	0.12
BMI (kg/m^2^)	27.38 ± 5.53	22.40 ± 4.23	**0.001**
FFM (kg)	54.25 ± 8.70	49.47 ± 6.27	**0.05**
FFMI (kg/m^2^)	19.75 ± 2.31	17.93 ± 1.94	**0.01**
ISWT (m)	221.78 ± 146.08	158.33 ± 88.63	0.10
ESWT (min)	6.06 ± 5.57	4.53 ± 5.22	0.09
SGRQ—Total score	55.45 ± 18.44	60.00 ± 18.67	0.09
SGRQ—Symptoms	55.24 ± 20.11	64.86 ± 17.77	0.08
SGRQ—Activity	70.95 ±23.55	78.01 ± 20.42	0.10
SGRQ—Impact	46.29 ± 19.16	47.83 ± 23.03	0.07
HAD—Depression	9.28 ± 1.90	8.77 ± 2.51	0.08
HAD—Anxiety	9.07 ± 2.63	8.66 ± 3.17	0.09

MRC: Medical research council; BMI: Body mass index; FM: Fat mass; FFM: Fat free mass; ISWT: Incremental shuttle walking test; ESWT: Endurance shuttle walking Test; SGRQ: St. George respiratory questionnaire; HAD scale: Hospital anxiety-depression scale;COPD: Chronic obstructive pulmonary disease; *P* values written in bold are statistically significant parameters

### Outcome measures of PR in early and late disease stages

**Figure 1:**
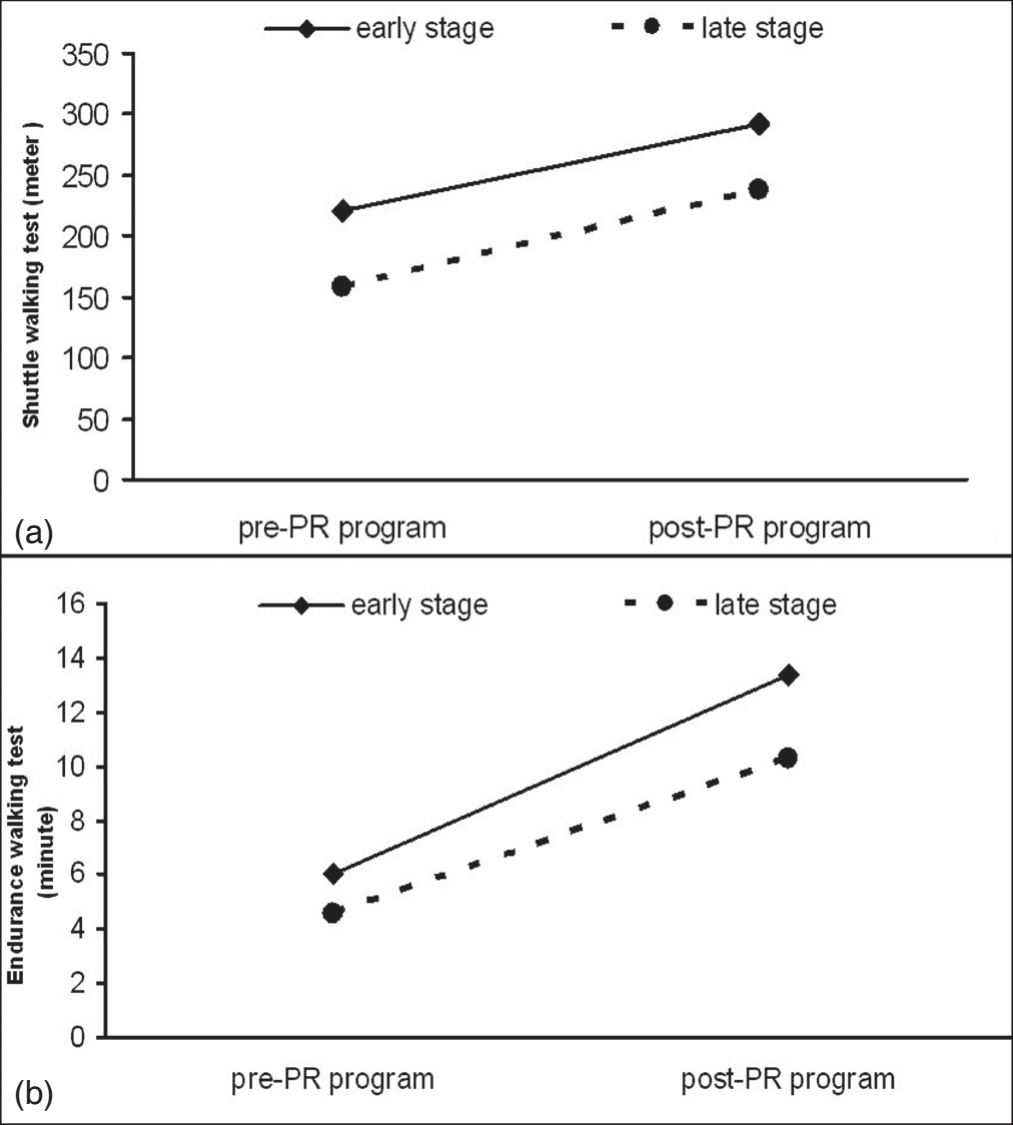
Early and late COPD groups showed statistically significant improvements in walking distance with ISWT (a) and the endurance time with ESWT (b) (*P* < 0.001) [Pre-PR: Before pulmonary rehabilitation, Post-PR: At the end of the pulmonary rehabilitation, incremental shuttle walking test (ISWT), endurance shuttle walking test (ESWT)]

**Figure 2:**
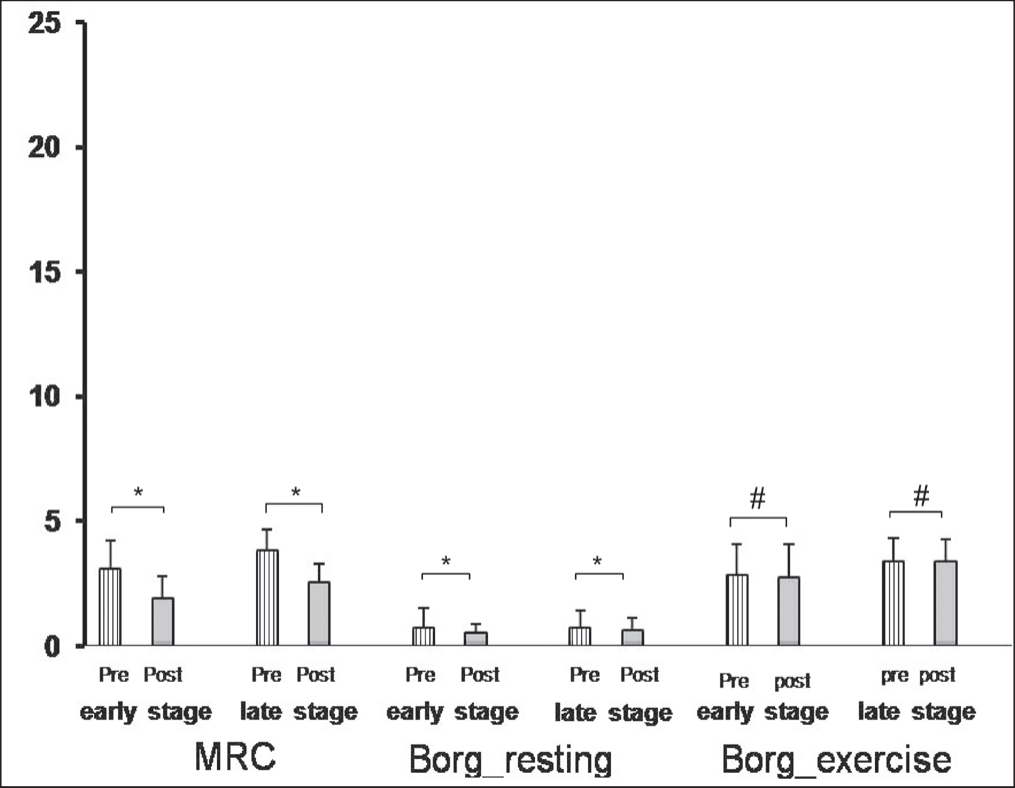
Statistically significant improvements both in early and late stages were shown in dyspnea sensation with MRC and resting BORG scores, but not in exercise BORG (Pre.: Before pulmonary rehabilitation, Post: At the end of the pulmonary rehabilitation, ^*^P < 0.05, ^#^*P* > 0.05)

**Figure 3:**
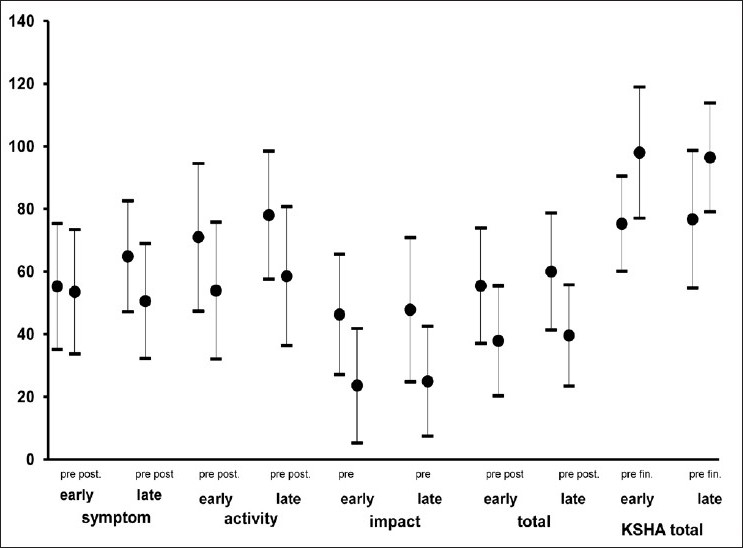
Health-related quality of life measurement with St. George Respiratory Questionnaire showed improvements in early and late COPD groups after pulmonary rehabilitation (*P* < 0.001), except symptom score domain in early stage (*P* > 0.05) (Pre.: Before pulmonary rehabilitation, Post.: At the end of the pulmonary rehabilitation)

**Figure 4:**
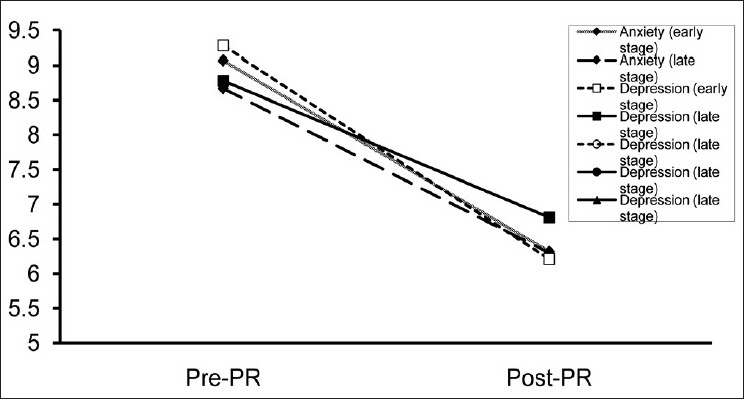
In both early and late disease stages, there was a statistically significant improvement for anxiety and depression scores with HAD questionnaire (*P* < 0.001)

The duration of PR program was 8 weeks. The effects of the PR program on study parameters were given in Table 2. At the end of the PR program, both FVC and FEV_1_had slightly improved but only in the early disease stage group the improvement in FVC had reached a statistically significant level. Exercise capacity (ISWT) [Figure 1a], exercise endurance time (ESWT) [Figure 1b], dyspnea sensation (MRC) [Figure 2], health-related quality of life (SGRQ total scores) [Figure 3] revealed an improvement in both early and late stage groups. For all of these parameters, the differences vs. baseline were statistically significant (*P* < 0.001). Although exertional dyspnea was reduced in both groups after PR, it was not found statistically significant [Figure 2]. Improvement in the SGRQ symptom domain only in the late stage group reached to a significant level (*P* < 0.001), whereas the difference in the early stage was not statistically significant. Activity and impact domains of SGRQ also improved after PR in both groups (*P* < 0.001) [Figure 3]. HAD scores were decreased significantly in both early and late disease stages (*P* < 0.001) [Figure 4].

**Table 2:** Variation of the principal parameters after rehabilitation (mean ± SD)

Parameters	Early stage (GOLD; I + II)	Late stage (GOLD; III + IV)
ΔFVC (% predicted)	5.17 ± 12.12*	0.96 ± 8.00#
ΔFEV_1_ (% predicted)	4.53 ± 12.39#	1.25 ± 4.93#
ΔMRC	−1.21 ± 0.73**	−1.25± 0.76**
ΔExercise—BORG	−0.08 ± 1.41#	−0.03 ± 1.12#
ΔBMI (kg/m^2^)	−0.20 ± 0.10#	0.20± 0.18#
ΔFFM (kg)	−0.52 ±2.20#	0.25 ±1.53#
ΔFFMI(kg/m^2^)	−0.18 ± 0.82#	0.06 ± 0.71#
ΔISWT(m)	71.42 ± 72.71**	80.18 ± 57.75**
ΔESWT (min)	7.33 ± 5.73**	5.77 ± 6.16**
ΔSGRQ—Total score	−17.55 ± 17.68**	−20.44 ± 17.06**
ΔSGRQ—Symptoms	−1.74 ± 15.81#	−14.30 ± 19.72**
ΔSGRQ—Activity	−17.03 ± 25.12**	−19.43 ± 21.56**
ΔSGRQ—Impact	−22.69 ± 19.80**	−22.87 ± 23.54**
ΔHAD—Depression	−3.07 ± 2.37**	−1.96 ± 3.01**
ΔHAD—Anxiety	−2.7 ± 2.6**	−2.3 ± 3.02**

FVC: Forced vital capacity; FEV_1_: Forced expiratory volume in the 1s; MRC: Medical Research Council Dyspnea Scale; BORG: BORG Dyspnea Scale; BMI: Body mass index; FFM: Fat Free Mass; FFMI: Fat free mass index; ISWT: Incremental shuttle walking test; ESWT: Endurance shuttle walking test; SGRQ: St. George’s Respiratory Questionnaire. (Reduction in scores of SGRQ indicates improvement, Reduction in scores of HAD indicates improvement).

* *P* < 0.05 vs. baseline.

** *P* < 0.001 vs. baseline

# *P*: NS vs. baseline

**Figure 5:**
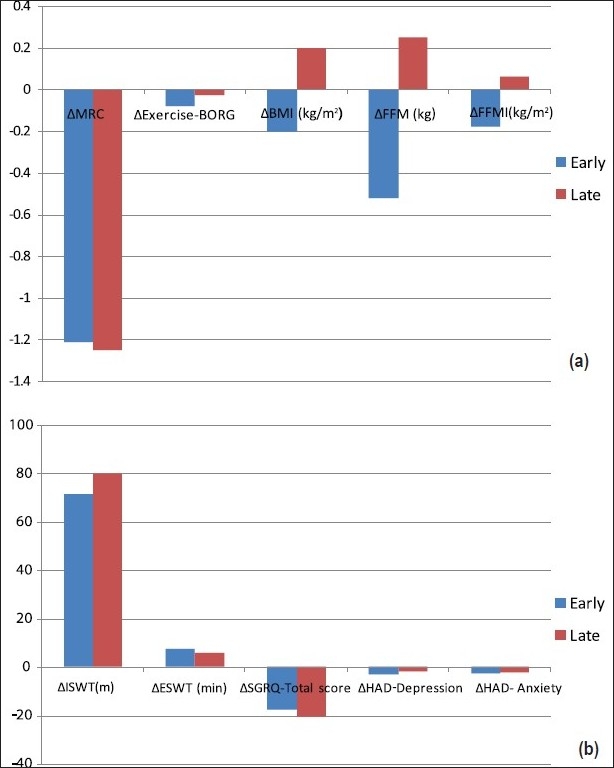
Gainings were statistically similar between early and late disease stage groups. (a) There were no significant difference in the case of improvement in MRC, BORG score, body mass index (BMI), fat free mass (FFM), and fat free mass index (FFMI) between early and late disease stage groups (*P* > 0.05). (b) There were no significant difference in the case of improvement in exercise capacity testing with incremental shuttle walkin test (ISWT), endurance shuttle walk test (ESWT), health-related quality of life (HQoL) with St. George Respiratory Questionnaire (SGRQ), and anxiety and depression scores with the HAD questionnaire between early and late disease stage groups (*P* > 0.05)

Gainings in the study parameters did not differ in the early and late disease stages [Figure 5]. In early and late disease stages, improvements in walking distance and endurance time were similar. FVC slightly improved in the early disease stage. This improvement was the average of 5.17% of predicted (*P* < 0.05) compared to 0.96% of predicted in the late disease stage (*P* > 0.05). The improvements in FEV_1_% did not differ between the groups (*P* > 0.05).

Dyspnea sensation scores evaluated with MRC decreased by 1.21 unit in the early stage group and by 1.25 unit in the late stage group (*P* = NS between groups). Similar but nonsignificant improvement was shown in exercise BORG scores.

There were significant differences between the groups after the rehabilitation in the meaning of body composition. In early stage, there were reductions in BMI, FFM, and FFMI (–0.20 ± 0.10 kg m^2^, –0.52 ± 2.20 kg, –0.18 ± 0.82 kg/m^2^, respectively) which were not significant according to the baseline. In the late disease stage, though there were increases in these parameters, they were not also significant (ΔBMI = 0.20 ± 0.18 kg/m^2^, ΔFFM = 0.25 ± 1.53 kg, ΔFFMI = 0.06 ± 0.71 kg/m^2^).

The distance walked during the ISWT showed a similar improvement in both groups, 71.42 m in the early disease stage and 80.18 m in the late disease stage groups (for both *P* = 0.001 vs. baseline). The improvement in endurance time also showed no difference (*P* = NS) between the two groups: 7.33 min (*P* = 0.001) in the earlier stage group and 5.77 min (*P* = 0.001) in the later one.

Also, health status for both groups was improved after rehabilitation. Improvement in the total score of SGRQ was –17.55 ± 17.68 unit in the early stage group, and –20.44±17.06 unit in the late stage group. These improvements were highly significant (*P* = 0.001 for each) and similar (*P* = NS) between the groups. In patients with the early disease stage, the decrease in the “symptoms” domain of SGRQ did not reach a significant level (-1.74 ± 15.81 unit, *P* = NS). On the other hand, the decrease in the late disease stage group was statistically significant (–14.30 ± 19.72 unit, *P* = 0.001). Scores of the “activity” and the “impact” domains showed a similar improvement in both groups. The decrement of these scores was statistically significant (*P* = 0.001, for each). In the late disease stage group for all scores of SGRQ, the improvement was also statistically significant, being >20 units for the “total”, >14 unit for the “symptom”, >19 unit for the “activity”, and >22 unit for the “impact” scores, well over the 4 unit threshold for clinical significance. In the early disease stage group except the “symptom” domain score, the improvements in the other domains of SGRQ were also over the minimal clinically significant level.

Also, psychometric assessments of the groups were similar after the rehabilitation (*P* = NS). Both depression and anxiety scores of HAD revealed an improvement (*P* = 0.001).

## Discussion

Chronic obstructive pulmonary disease is a disease that is not confined to airways and the lungs, but also produces systemic consequences so a multidisciplinary approach must be taken into account. Pulmonary rehabilitation is recognized as a cornerstone of COPD treatment: it ameliorates symptoms and exercise capacity, improving health-related quality of life.[1–3,8]

A comprehensive PR program for COPD patients includes: patient assessment; exercise training, education of the patient and family, nutritional, psychosocial counseling, and support.[3,8,12] Although GOLD 2009 recommended PR for COPD patients from stage II (FEV_1_< 80%), physician referrals to this intervention generally include late stages of the disease.[1,11,12] In a recent study, it was shown that GOLD classification can be used to discern groups of COPD patients, but due to large inter-individual variability it does not seem adequate as a basis for individual management plans in rehabilitation. Though, there were little researches specifically targeted for early disease stages (GOLD I and II). Berry *et al*.[13] showed that exercise training alone improved physical function in patients with COPD at all stages. In this study, the group with mild disease now corresponding to stage II according to GOLD 2009 classification was the largest number of patients compared with the severe and very severe groups. Takigawa *et al*.[3] also showed that COPD patients with an FEV_1_< 80% (stages II, III, and IV) made gains in physical function with a 4–8 week PR program though the study population was the opposite of Berry’s study. Therefore, it is possible to say that the results of them much more relevant with the outcomes of late disease stages (GOLD III and IV).[3,13] In this study, our primary aim was to evaluate the benefit of a comprehensive pulmonary rehabilitation program composed of exercise training, nutritional and psychosocial counseling and educational sessions in an hospital based, outpatient setting and the secondary aim was to compare the outcomes of PR in the early stage with the late stage of COPD. We grouped the patients as early and late stages as there were only two patients who were GOLD stage I and four patients who were GOLD stage IV. Although it was not possible to say that this study fully representative for discriminating the effects of PR according to the GOLD stages, it will address the need of a multidisciplinary intervention for patients with GOLD stage II also. The baseline characteristics of the study population revealed a considerable similarity between early and late stages for the impairments in exercise capacity, HRQoL, and psychological status. These results are in accordance with the studies impressing that the patients in GOLD stage II might have similar impairments in exercise capacity and HRQoL in stage IV.[3,13]

Being a systemic disease, abnormalities in body composition are another important event of COPD. The baseline characteristics of the patients according to the body composition parameters were different in this study. Although the difference was not significant and could be accepted in the normal range, BMI, FFM, and FFMI of the patients in the late stage of disease were lower than the patients with the early stage in our study. Body mass index and FFMI represent different aspects of nutritional abnormalities in COPD and in previous studies, it has been already shown that low FFMI is not only correlated well with the severity of COPD, exercise tolerance but also with the survival.[16,23,24] Therefore, assessment of body composition should be taken into account in the management of COPD patients. Apart from physical impairment, patients with COPD carry substantial mental burden related to their disease and its symptoms. Funk *et al*.[23] mentioned that anxious and depressive symptoms are common in the patients with advanced COPD. In our study, we saw that HAD scores of the patients in early and late stages of disease were similar. In Funk’s study, anxious symptoms were explained by dyspnea, on the other hand depressive symptoms were explained by both dyspnea and reduced exercise capacity.[23] Although this subject was not taken into account in the present study, we are evaluating whether there is any relation with psychosocial symptoms, HRQoL and exercise capacity in COPD patients in another study which will be published in future.

In our study, all patients were admitted to a hospital based, out-patient PR unit, two hours per a day, and two days per week. The Turkey National Health System does not routinely reimburse all costs in this area and our center is the unique one, set to manage a multidisciplinary comprehensive PR program.

### Lung function

At the end of the PR, both FVC and FEV_1_ slightly improved in both groups but only in the early disease stage group the improvement in FVC% of predicted reached a statistically significant level. As some of the patients in this study could not cooperate with carbon monoxide diffusion capacity testing, results of the others also could not be taken into consideration. Although we do not have any clear explanation why the FVC improved significantly only in earlier stages, it may be related with the high degree of hyperinflation in the late disease stages. This result was in accordance with Berry’s which especially evaluated early stages of COPD and showed an improvement of pulmonary function.[13] In Takigawa’s study significant improvements in FEV_1_% of predicted and % of residual volume in stages III and IV, in % vital capacity in stages II, III, IV, and % of total lung capacity in stage II after PR.[3]

During our comprehensive PR program, adherence to prescribed therapy and medication advice were the main topics of the patient education sessions. Therefore, another possible explanation of the improvement in pulmonary function will be based on the treatment compliance.

### Symptoms

Medical Research Council Scale is simple to administer for evaluating dyspnea during everyday activities.[1,16] In this study, dyspnea sensation evaluated with MRC significantly decreased both in the early and the late disease stage groups. There was not any difference in the improvement of the dyspnea sensation level between the groups. We used the modified BORG Scale for testing exertional dyspnea. After PR, similar but nonsignificant decreases in scores of BORG were shown in early and late stage groups. To us it is important to note that COPD patients with the GOLD stage II, can also be symptomatic and encouraging them to participate in a PR so crucial.

### Nutritional status

Nutritional screening and therapy are considered an essential component of integrated COPD management. The association between underweight and increased mortality risk has been well established in numerous retrospective studies.[16,23,24] Studies related to body compositions have shown that weight loss is accompanied by significant loss of fat-free mass that is related to impaired skeletal muscle strength and exercise capacity in patients with COPD.[1,8,25,26] These studies have furthermore shown that muscle wasting may also occur in normal weight subjects.[27–29] In our study, when the basal characteristics of the nutritional status in the early-stage group checked, there were not any patient who were normal or over weight but with a FFMI <17 kg/m^2^. Therefore, these patients were not supported with any oral supplement but given dietary advices. However, after a 8-week PR program, BMI, FFM, and FFMI of the early COPD group revealed statistically nonsignificant decrease. These results suggested that exercise training resulted in negative energy balance, so as it was shown in the previous studies in selected patients, especially in patients with normal BMI, prescribing oral nutritional supplement may overcome this problem.[30,31] In the late stage group, the patients with the BMI ≤ 19 and FFMI < 17 kg/m^2^ were given oral nutritional support during the PR program. In this group of patients, no any reductions in BMI, FFM, and FFMI were seen.

### Exercise tolerance

In this study, while evaluating the exercise capacity and prescribing endurance training, ISWT and ESWT were used. The distance walked during the ISWT showed a similar improvement in the two groups of patients (71.42 ± 72.71 m in early stage and 80.18±57.75 m in the late stage group). In this regard, the increments for the two groups were exceed the threshold considered significant for a clinically significant improvement which was shown as 48 m when assessed at a population level for ISWT.[32] The ESWT is a simple, acceptable and highly responsive outcome measure for COPD patients undergoing a pulmonary rehabilitation program.[33,34] Being a variant of the SWT, it was designed as an alternative to SWT and 6 min walking test that would better reflect the submaximal exertion that individuals use in performance of their daily activities.[34,35] In our study, the improvement in endurance time was also statistically significant and there were no any statistically meaningful difference in the gainings between the groups.

In conclusion, our study demonstrates positive outcomes of a comprehensive PR program in COPD patients at all stages of the disease. So just like the patients with later disease stage, the patients with earlier diseases stages, such as GOLD stage I and stage II, should be encouraged to ensure awareness of the disease, to improve their HRQoL, psychological status, and exercise capacity.
